# Predictors of prolonged mechanical ventilation after surgery for hypertensive basal ganglia intracerebral hemorrhage: a retrospective cohort study

**DOI:** 10.3389/fmed.2026.1797872

**Published:** 2026-04-09

**Authors:** Guangwei Xie, Zhongling Yang, Dongdong Zou, Yifan Jiang, Shuping Chen, Lin He, Yan Zou

**Affiliations:** 1 Shanghai Sixth People’s Hospital Affiliated to Shanghai Jiao Tong University School of Medicine, Shanghai, China; 2 Shanghai General Hospital, Shanghai, China

**Keywords:** hypertensive basal ganglia hemorrhage, predictive model, prolonged mechanical ventilation, retrospective cohort study, risk factors

## Abstract

**Background:**

Spontaneous intracerebral hemorrhage (SICH), particularly hypertensive basal ganglia hemorrhage, is a severe stroke subtype associated with high mortality and disability. A substantial proportion of these patients require mechanical ventilation (MV), and prolonged MV (PMV) is associated with increased complications and worse outcomes. While certain clinical and radiological factors have been linked to PMV in general critical care or stroke populations, evidence specific to patients with hypertensive basal ganglia hemorrhage remains scarce, limiting early risk stratification.

**Objective:**

To determine the incidence of PMV (≥14 days of MV) and identify independent predictors in postoperative patients with hypertensive basal ganglia SICH.

**Methods:**

This retrospective cohort study (September 2019 to September 2025) included 173 consecutive adult patients with hypertensive basal ganglia SICH who underwent surgery and required postoperative MV. PMV was defined as MV lasting ≥14 days from intensive care unit (ICU) admission. Potential predictors were screened using univariate analyses and entered into multivariable logistic regression to identify independent predictors. Model discrimination and calibration were assessed.

**Results:**

Among 173 postoperative patients with hypertensive basal ganglia hemorrhage requiring MV, PMV (≥14 days) occurred in 55 (31.8%). In multivariable logistic regression, older age (adjusted OR 1.05 per year, 95% CI 1.02–1.07), chronic kidney disease (adjusted OR 5.46, 95% CI 1.91–18.43), lower admission Glasgow Coma Scale (GCS) score (adjusted OR 0.82 per point, 95% CI 0.72–0.91), and intraoperative drainage catheter placement (adjusted OR 3.51, 95% CI 1.91–6.64) were independent predictors of PMV. A prediction model incorporating these variables showed moderate-to-good discrimination (AUC 0.779, 95% CI 0.702–0.856) and acceptable calibration.

**Conclusion:**

PMV (≥14 days) affected approximately one-third of critically ill postoperative patients with hypertensive basal ganglia hemorrhage. A model based on age, chronic kidney disease, admission GCS score, and intraoperative drainage catheter placement demonstrated moderate-to-good discrimination and acceptable calibration, and may support early postoperative risk stratification; external validation is warranted before routine clinical implementation.

## 1 Introduction

Spontaneous intracerebral hemorrhage (SICH) is one of the most devastating subtypes of stroke in terms of mortality and disability, accounting for approximately 10%–30% of all strokes (1). SICH clinical features, etiology, and early prognosis differ markedly by hematoma location. Lobar ICH has a distinct clinical profile and worse early prognosis than deep subcortical ICH (in-hospital mortality 26.7% vs. 16.5%), with non-hypertensive mechanisms predominating in lobar ICH, while hypertensive vasculopathy is the primary cause of deep ICH, most often affecting the basal ganglia (1). In hypertensive intracerebral hemorrhage, the basal ganglia are among the most common sites of hemorrhage; deep-seated hematomas are frequently associated with severe neurological deficits, secondary cerebral edema/intracranial hypertension, and impaired consciousness (2–4), placing higher demands on airway protection and respiratory support management during the perioperative and intensive care phases (5–7). Approximately 17%–33% of patients with acute central nervous system injury require endotracheal intubation and mechanical ventilation (8), and more than 10% of stroke patients also need mechanical ventilation support (9). Prolonged mechanical ventilation (PMV) is closely associated with ventilator-associated complications, prolonged hospital stay, and poor outcomes (10, 11). Previous studies have indicated that advanced age, obesity, chronic obstructive pulmonary disease, atrial fibrillation, and severe comorbidities are related to PMV in critically ill patients (12, 13); In patients with hemorrhagic stroke, larger hematoma volume, obstructive hydrocephalus, coma or brain herniation, hemorrhage location (e.g., thalamus), and elevated inflammatory and immune-related markers may also increase the risk of PMV (14, 15). As confirmed by Passero et al., intraventricular extension (IVE) is an independent determinant of poor ICH outcomes, as it exacerbates intracranial hypertension, hydrocephalus, and consciousness impairment, and is thus closely correlated with an increased risk of PMV (2). However, studies on PMV in patients with intracerebral hemorrhage remain limited overall, and evidence specifically focusing on hypertensive basal ganglia hemorrhage is even scarcer, making it difficult for clinicians to perform reliable early risk stratification and resource allocation.

Accordingly, this study aimed to determine the incidence of PMV (defined as mechanical ventilation lasting ≥14 days) and to identify independent predictors among patients with hypertensive basal ganglia SICH. We hypothesized that older age, worse neurological status at admission, greater hemorrhage burden, and elevated inflammatory and immune-related markers would be associated with a higher risk of PMV.

## 2 Methods

### 2.1 Research design

A retrospective cohort study was conducted, enrolling consecutive patients who underwent surgery for basal ganglia SICH at the Sixth People’s Hospital Affiliated to Shanghai Jiao Tong University. All participants required postoperative mechanical ventilation and were admitted to the intensive care unit (ICU) for ≥24 h. The primary outcome was PMV, defined as a postoperative mechanical ventilation duration—from initiation after ICU transfer to successful weaning—of ≥14 days. Successful weaning was defined as no reinitiation of mechanical ventilation for ≥48 consecutive hours after ventilation cessation; if reintubation or re-ventilation occurred, the time of final successful weaning was used. Eligible cases were identified between September 2019 and September 2025. Data were extracted from electronic medical records, anesthesia and surgical documentation, ICU ventilator and nursing records, laboratory systems, and imaging systems. This study was conducted in accordance with the Declaration of Helsinki and was approved by the Ethics Committee of Shanghai Sixth People’s Hospital [Approval No.: 2024-KY-128(K)]. Written informed consent was obtained from the participants or their legal guardians.

### 2.2 Participants

Patients who underwent surgery for basal ganglia intracerebral hemorrhage at the Sixth People’s Hospital Affiliated to Shanghai Jiao Tong University between September 2019 and September 2025 were included. Inclusion criteria were: (1) age ≥18 years; (2) basal ganglia intracerebral hemorrhage confirmed by cranial computed tomography (CT) or magnetic resonance imaging (MRI) and treated surgically; (3) postoperative mechanical ventilation with ICU stay ≥24 h. Exclusion criteria were: (1) age >80 years or preoperative Glasgow Coma Scale (GCS) score <5; (2) severe pre-existing respiratory disease (e.g., chronic respiratory failure, long-term oxygen therapy, or recurrent acute exacerbations); (3) death or discharge within 14 days after surgery; (4) missing key data (unable to determine ventilation duration or major candidate variables). No individual matching was performed; potential confounding was addressed using multivariable models.

### 2.3 Surgical treatment

Upon patients’ arrival at the emergency department, attending physicians performed immediate cranial CT following initial clinical assessment. Hematoma volume in intracerebral hemorrhage was estimated using the ABC/2 method: A denotes the maximum diameter of the hematoma on the largest CT slice, B represents the maximum diameter perpendicular to A on the same slice, and C is calculated as the approximate number of slices containing the hematoma multiplied by the slice thickness. All eligible patients underwent surgical intervention. Operative procedures were classified into four mutually exclusive categories according to the principal surgical approach: cranial puncture drainage, endoscopic hematoma evacuation, craniotomy without decompressive craniectomy, and craniotomy with decompressive craniectomy. Microscope assistance, when used, was considered a technical adjunct within open craniotomy rather than a separate operative category. Intraoperative drainage catheter placement was recorded as a separate surgery-related variable and was not included in the operative classification. Decisions regarding intraoperative drainage catheter placement or external ventricular drainage (EVD) were made by integrating the GCS score, pupillary findings, CT results, age, hematoma volume and location, and signs of neurological deterioration. Imaging variables were obtained from formal reports and original CT or MRI images; original images were reviewed for verification when deemed necessary.

### 2.4 Admission to the ICU for treatment

All patients were transferred to the ICU immediately following surgery. Postoperative management encompassed sedation, analgesia, mechanical ventilation, intracranial pressure (ICP) monitoring, mannitol therapy for intracranial hypertension, acid suppression and gastric mucosal protection, antiepileptic agents (sodium valproate or levetiracetam), antibiotic prophylaxis for at least 3 days, and enteral nutrition via a nasogastric tube. Standard ICU monitoring included continuous invasive arterial blood pressure monitoring and pulse oximetry. Neurological assessments were performed hourly, including GCS scoring, pupillary size, and light reflexes. A repeat cranial CT scan was performed on postoperative day 2; urgent reimaging was undertaken if ICP exceeded 20 mmHg or new unilateral or bilateral mydriasis was observed.

### 2.5 Management of mechanical ventilation

All patients received mechanical ventilation support upon admission to the ICU. After the patient’s condition stabilized, sedation was discontinued every early morning to assess the level of consciousness and weaning conditions. Patients who met the weaning criteria underwent a spontaneous breathing trial (SBT), and extubation was evaluated after successful completion of the SBT. Percutaneous tracheostomy was performed in patients with weaning failure, those expected to require mechanical ventilation support for ≥2 weeks, or those with extubation failure. After tracheostomy, the daily weaning process was repeated continuously on the basis of stable condition until complete weaning from mechanical ventilation was achieved.

SBT protocol: The ventilator was disconnected, and oxygen was administered via a T-piece at 3 L/min. The SBT was terminated if any of the following occurred: (1) rapid shallow breathing index (RSBI) > 105; (2) respiratory rate >35 breaths/min or <8 breaths/min; (3) heart rate >140 beats/min, a > 20% change from baseline, or new-onset arrhythmia. After a 3-min screening period, spontaneous breathing was continued for an additional 30 min; the SBT was considered successful if the patient tolerated the trial. All key ventilation-related outcomes and time points were extracted from ICU ventilator records, medical orders, and nursing records, with cross-checking when necessary.

### 2.6 Statistical analysis

Patients were divided into two groups according to the occurrence of PMV, (≥14 days). Continuous variables were presented as mean ± standard deviation or median (interquartile range) based on their distribution, while categorical variables were reported as number (%). Univariate logistic regression analysis was used to initially screen candidate factors associated with PMV. Variables with a univariate *p* < 0.05 were included in a multivariate logistic regression model to identify independent risk factors, with adjustment for key confounding factors based on clinical relevance. For the final multivariable logistic regression model, diagnostic performance at the optimal cutoff identified from the ROC curve was additionally evaluated using sensitivity, specificity, positive predictive value (PPV), negative predictive value (NPV), and overall accuracy. Continuous variables were preferentially entered into the model in their continuous form, and adjusted odds ratios (OR) with 95% confidence intervals (CI) were reported.

Regarding missing data, the missing proportion of each variable was first described. Patients with missing key outcome data had been excluded in the inclusion and exclusion criteria; for the remaining variables, complete case analysis was primarily adopted, and multiple imputation was used as a sensitivity analysis to evaluate the robustness of the results. All tests were two-tailed, and a *p*-value < 0.05 was considered statistically significant. Statistical analyses were performed using *R* statistical software.

## 3 Results

### 3.1 Participant flow and baseline characteristics

**Figure 1:**
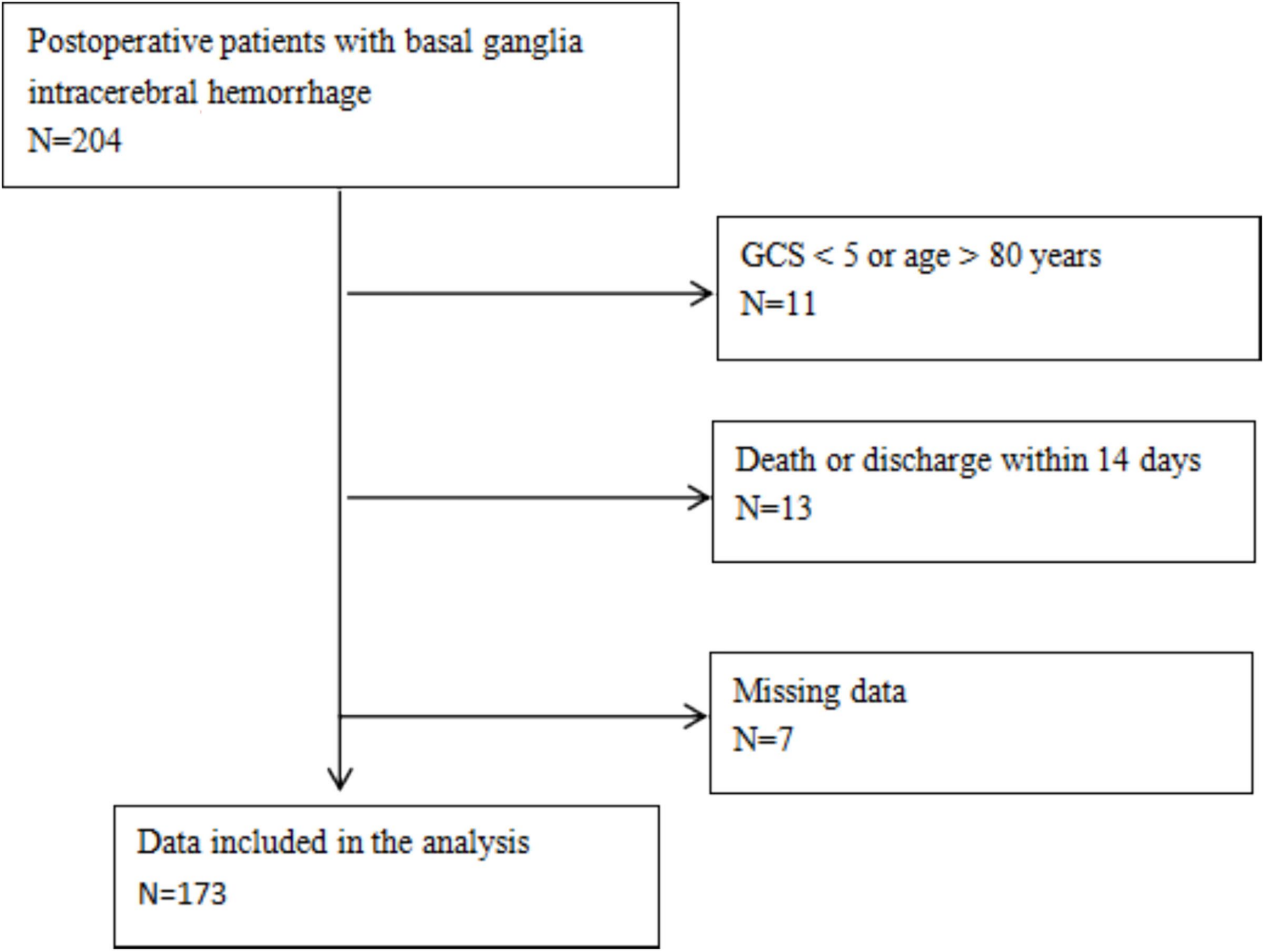
Flow diagram of patient enrollment.

Of the 204 initially screened patients, 11 were excluded due to a GCS score <5 or age >80 years; 13 died or were discharged against medical advice within 14 days; and 7 were excluded due to incomplete data. Finally, 173 eligible patients were included in the analysis. The mean age was 52.9 ± 12.1 years, and 147 (85.0%) were male. Tracheostomy was performed in 104 (60.1%) patients, and the median duration of mechanical ventilation was 10 days (IQR, 6–15) (Figure 1).

The median admission GCS score was 8 (IQR, 7–11), and preoperative aspiration was present in 34 (19.7%) patients. Regarding hemorrhage characteristics, 72 (41.6%) patients had left-sided hematoma, 34 (19.7%) had preoperative brain herniation, 109 (63.0%) had intraventricular extension, and the median hematoma volume was 40 mL (IQR, 27–62). Intraoperative drainage catheter placement was performed in 100 (57.8%) patients. According to the revised operative classification, 32 (18.5%) patients underwent cranial puncture drainage, 56 (32.4%) underwent endoscopic hematoma evacuation, 45 (26.0%) underwent craniotomy without decompressive craniectomy, and 40 (23.1%) underwent craniotomy with decompressive craniectomy. The baseline characteristics of the study population are summarized in Table 1.

**Table 1:** Baseline characteristics of postoperative patients with hypertensive basal ganglia intracerebral hemorrhage (*n* = 173).

Variable	Total (*n* = 173)
Age, years	52.9 ± 12.1
Male sex, [*n* (%)]	147 (85.0)
Body mass index, kg/m^2^	26.2 ± 3.8
Chronic heart disease, [*n* (%)]	11 (6.4)
Diabetes mellitus, [*n* (%)]	19 (11.0)
Chronic kidney disease, [*n* (%)]	14 (8.1)
Admission GCS score, median (IQR)	8 (7.0, 11.0)
Preoperative aspiration, [*n* (%)]	34 (19.7)
Tracheostomy, [*n* (%)]	104 (60.1)
Mechanical ventilation duration, days, median (IQR)	10 (6.0, 15.0)
Left-sided hematoma, [*n* (%)]	72 (41.6)
Preoperative brain herniation, [*n* (%)]	34 (19.7)
Intraventricular extension, [*n* (%)]	109 (63.0)
Hematoma volume, mL, median (IQR)	40 (27.0, 62.0)
Intraoperative drainage catheter placement [*n* (%)]	100 (57.8)
Cranial puncture drainage	32 (18.5)
Endoscopic hematoma evacuation	56 (32.4)
Craniotomy without decompressive craniectomy	45 (26.0)
Craniotomy with decompressive craniectomy	40 (23.1)

Values are presented as mean ± SD, median (IQR), or n (%). BMI, body mass index; GCS, Glasgow Coma Scale; IQR, interquartile range.

### 3.2 Comparison of baseline characteristics between the non-PMV and PMV groups

Comparisons between the non-prolonged mechanical ventilation group and the prolonged mechanical ventilation group were performed to examine differences in baseline clinical characteristics, hemorrhage-related variables, surgery-related variables, and laboratory parameters. The results showed that age, chronic kidney disease, admission GCS score, preoperative brain herniation, hematoma volume, intraoperative drainage catheter placement, and procalcitonin level differed significantly between the two groups (Table 2). In-hospital mortality within the analytic cohort was 9.2% (16/173) and was significantly higher in the PMV group than in the non-PMV group (10/55, 18.2% vs. 6/118, 5.1%; *p* = 0.006). Because patients who died or were discharged within 14 days after surgery were excluded by design, these mortality estimates apply only to the analytic cohort.

**Table 2:** Comparison of baseline characteristics between patients without prolonged mechanical ventilation and those with prolonged mechanical ventilation.

Variable	Non-prolonged mechanical ventilation group	Prolonged mechanical ventilation group	Test statistic (*t*/*Z*/*χ*^2^)	*p*-value
Age (years)	50.91 ± 11.26	57.13 ± 12.96	−3.223	0.002
Gender [*n* (%)]			0.015	0.903
Female	18 (15.25)	8 (14.55)		
Male	100 (84.75)	47 (85.45)		
Body Mass Index (BMI, kg/m^2^)	26.12 ± 3.76	26.42 ± 3.98	−0.486	0.628
Heart disease [*n* (%)]			0.450	0.502
None	112.00 (94.92)	50.00 (90.91)		
Yes	6.00 (5.08)	5.00 (9.09)		
Diabetes mellitus [n (%)]			0.251	0.616
None	106 (89.83)	48 (87.27)		
Yes	12 (10.17)	7 (12.73)		
Kidney disease [n (%)]			5.876	0.015
None	113 (95.76)	46 (83.64)		
Yes	5 (4.24)	9 (16.36)		
Admission Glasgow Coma Scale (GCS) score (points)	9.00 (8.00, 11.00)	8.00 (5.50, 9.00)	3.838	<0.001
Aspiration [n (%)]			0.111	0.740
None	94 (79.66)	45 (81.82)		
Yes	24 (20.34)	10 (18.18)		
Location of intracerebral hemorrhage [n (%)]			0.392	0.531
Left	51 (43.22)	21 (38.18)		
Right	67 (56.78)	34 (61.82)		
Brain herniation [n (%)]			6.470	0.011
None	101 (85.59)	38 (69.09)		
Yes	17 (14.41)	17 (30.91)		
Hematoma volume (mL)	35.30 (25.00, 55.92)	45.60 (35.50, 68.00)	−2.737	0.006
Surgical Technique (*n*%)			5.814	0.121
Cranial puncture drainage	19 (16.10)	13 (23.64)		
Endoscopic hematoma evacuation	41 (34.75)	15 (27.27)		
Craniotomy without decompressive craniectomy	35 (29.66)	10 (18.18)		
Craniotomy with decompressive craniectomy	23 (19.50)	17 (30.90)		
Intraoperative drainage catheter placement [n (%)]			11.388	0.001
None	60 (50.85)	13 (23.64)		
Yes	58 (49.15)	42 (76.36)		
Tracheotomy [n (%)]			2.851	0.091
None	42 (35.59)	27 (49.09)		
Yes	76 (64.41)	28 (50.91)		
PaO₂/FiO₂, (mmHg)	317.59 ± 106.52	332.55 ± 101.85	−0.872	0.385
WBC, (×10^9^/L)	10.17 ± 3.21	10.90 ± 3.91	−1.294	0.198
Neutrophil-to-lymphocyte ratio (NLR)	6.95 (4.19, 11.45)	8.50 (3.85, 14.06)	−0.603	0.548
Hb (g/L)	130.84 ± 16.84	129.91 ± 19.94	0.319	0.750
PLT (×10^9^/L)	193.47 ± 62.74	197.73 ± 65.67	−0.410	0.682
MPV (fL)	10.51 ± 0.98	10.61 ± 0.96	−0.619	0.537
CRP(mg/L)	1.90 (0.13, 8.55)	3.39 (0.58, 15.55)	−1.692	0.091
GLU (mmol/L)	7.65 (6.43, 8.90)	7.60 (6.80, 9.75)	−1.897	0.058
Lac (mmol/L)	2.20 (1.40, 3.08)	2.30 (1.60, 3.25)	−0.735	0.463
ALB (g/L)	35.60 (32.00, 40.90)	38.00 (31.25, 44.45)	−1.092	0.275
PCT (ng/mL)	0.07 (0.04, 0.16)	0.15 (0.07, 0.28)	−2.808	0.005
NT-proBNP, (pg/mL)	129.50 (60.83, 254.90)	169.00 (79.35, 473.00)	−1.842	0.066
In-hospital mortality, *n* (%)	6 (5.1)	10 (18.2)	7.667	0.006

Continuous data are presented as mean ± standard deviation or median (interquartile range); categorical data are expressed as *n* (%). Comparisons between groups were performed using the t-test or Mann–Whitney *U* test for continuous data, and the *χ*^2^ test or Fisher’s exact test for categorical data. BMI, GCS, WBC, NLR, Hb, PLT, MPV, CRP, PCT, NT-proBNP.

### 3.3 Multivariable logistic regression analysis of independent predictors of prolonged mechanical ventilation

Variables that were statistically significant in the univariable analyses were entered into a multivariable logistic regression model with stepwise selection. Age, chronic kidney disease, admission GCS score, and intraoperative drainage catheter placement remained independent predictors of prolonged mechanical ventilation (Table 3).

**Table 3:** Multivariate analysis of prolonged mechanical ventilation.

Variables	B	S. E.	z	*p*	OR (95% CI)
Age(years)	0.045	0.013	3.454	0.001	1.05 (1.02–1.07)
Chronic kidney disease	1.697	0.569	2.984	0.003	5.46 (1.91–18.43)
GCS score	−0.203	0.059	−3.437	0.001	0.82 (0.72–0.91)
Intraoperative drainage catheter placement	1.256	0.317	3.961	<0.001	3.51 (1.91–6.64)
Constant	−1.651	0.969	−1.704	0.088	

Because catheter placement is inherent to cranial puncture drainage, we further examined the distribution of intraoperative drainage catheter placement across surgical classifications and performed a sensitivity analysis excluding patients who underwent cranial puncture drainage. Catheter placement was procedure-dependent, occurring in 100.0% of cranial puncture drainage cases and varying across the other operative categories. In the restricted cohort, intraoperative drainage catheter placement remained independently associated with prolonged mechanical ventilation after adjustment for age, chronic kidney disease, admission GCS score, and surgical classification (OR 2.92, 95% CI 1.37–6.24; *p* = 0.006), whereas surgical classification was not independently associated with prolonged mechanical ventilation (overall *p* = 0.148) (Supplementary Tables S1–S3).

### 3.4 Evaluation of predictive model discrimination and calibration

**Figure 2:**
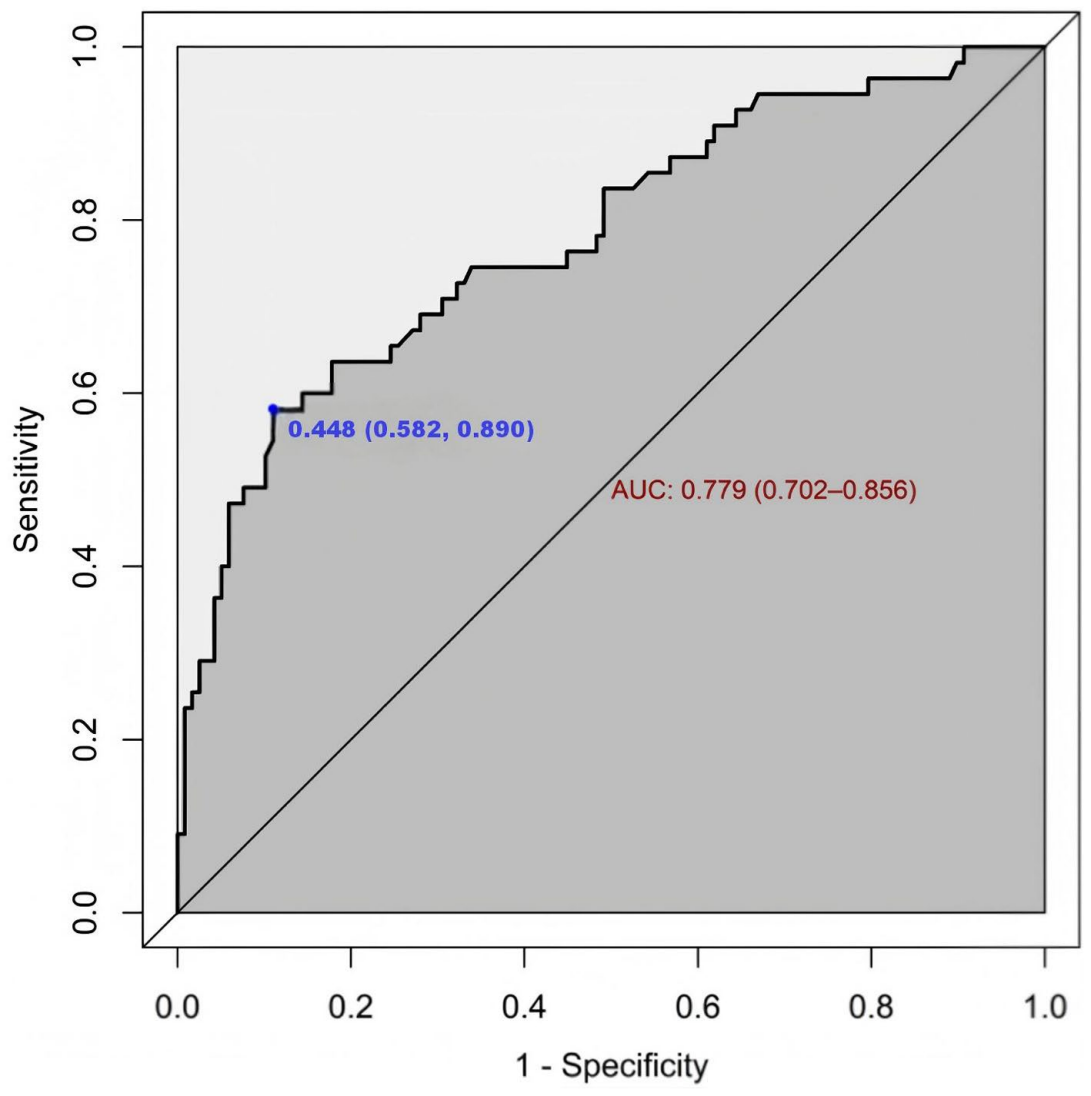
Receiver operating characteristic (ROC) curve for the predictive model of prolonged mechanical ventilation.

The model yielded an AUC of 0.779 (95% CI, 0.702–0.856), indicating moderate-to-good discrimination for prolonged mechanical ventilation (Figure 2). The optimal cutoff value determined from the ROC curve was 0.448. At this threshold, the sensitivity, specificity, positive predictive value, negative predictive value, and overall accuracy were 0.582, 0.890, 0.711, 0.820, and 0.792, respectively (Table 4).

**Table 4:** Diagnostic performance of the final multivariable logistic regression model for prolonged mechanical ventilation.

Model	Cutoff	AUC	Accuracy	Sensitivity	Specificity	PPV	NPV
Final multivariable model	0.448	0.779	0.792	0.582	0.89	0.711	0.82

**Figure 3:**
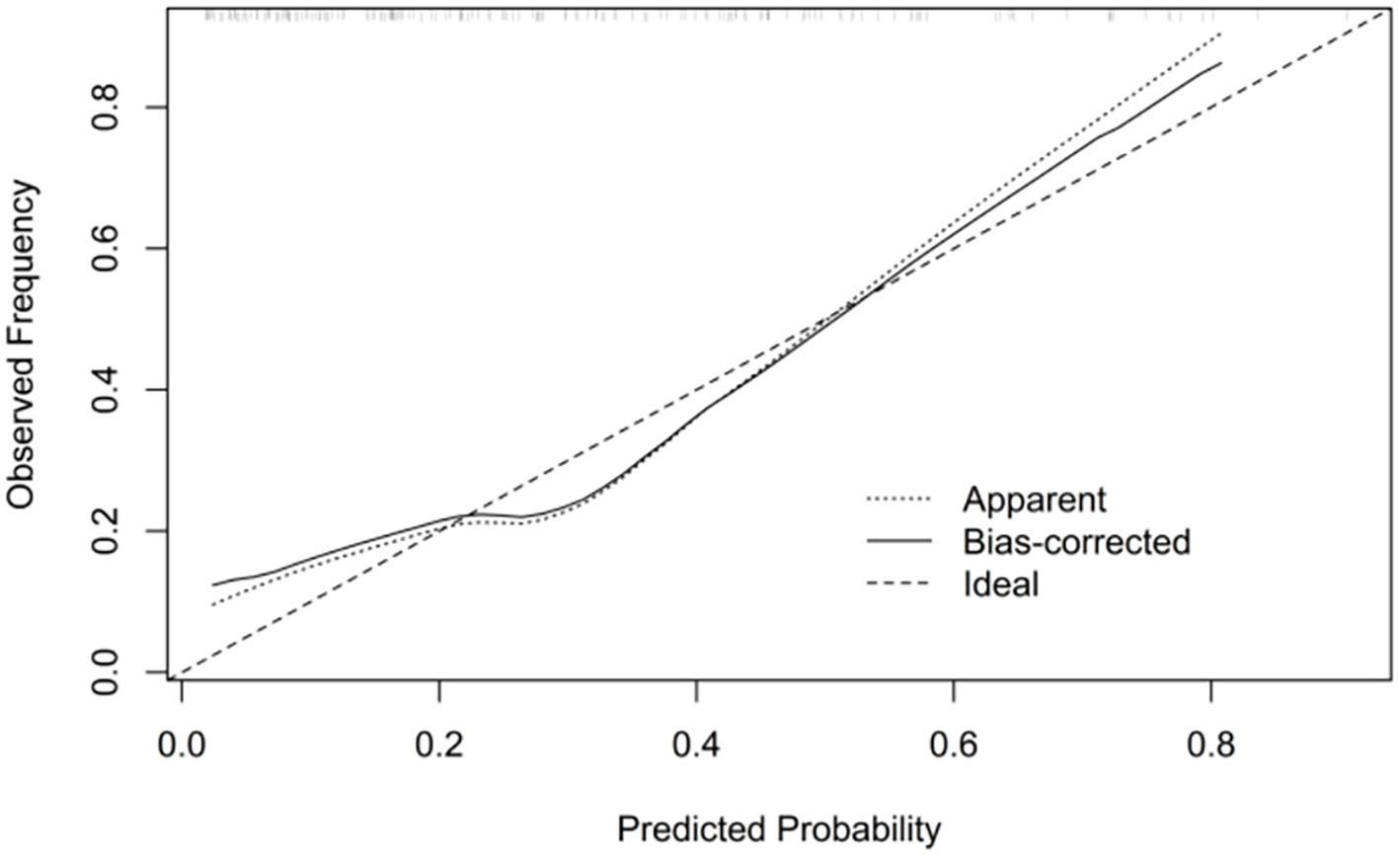
Calibration plot of the predictive model for prolonged mechanical ventilation.

Calibration was assessed using a calibration plot. The plot demonstrated overall agreement between predicted probabilities and observed incidence; the bias-corrected curve was close to the ideal line, suggesting acceptable calibration after internal validation (Figure 3).

## 4 Discussion

To our knowledge, evidence focusing specifically on neurosurgical postoperative patients with basal ganglia intracerebral hemorrhage remains limited. In this study, we investigated risk factors for PMV and developed a prediction model using readily available preoperative and intraoperative variables. Four independent predictors were identified: older age, comorbid chronic kidney disease, lower admission GCS score, and intraoperative drainage catheter placement. The model showed moderate-to-good discrimination (AUC = 0.779), which may help identify high-risk patients early and support targeted management.

The basal ganglia are adjacent to critical neural conduction pathways such as the internal capsule and corticospinal tract. Hemorrhage in this region not only causes severe motor dysfunction but may also affect the thalamic–brainstem arousal network via hematoma compression and secondary edema, thereby impairing consciousness and weakening airway protective capacity earlier and more severely (3). In the present study, the incidence of PMV in postoperative patients with basal ganglia intracerebral hemorrhage was 31.8%. In comparison, a review by Huang et al. (4) reported that approximately 5%–13% of patients with acute respiratory failure require PMV. This discrepancy suggests that critically ill neurosurgical patients, particularly those with injury in specific brain regions, may have a higher risk of prolonged ventilation and more complex weaning challenges.

Each 1-year increase in age was associated with an approximately 5% higher risk of PMV (OR = 1.05). This finding is consistent with reduced physiologic reserve and poorer tolerance of weaning in older patients, including lower cardiopulmonary reserve and respiratory muscle strength, weaker cough and swallowing reflexes, slower clearance of sedatives/analgesics, higher susceptibility to infection, and delayed early neurological recovery. Similarly, Ho et al. (5) identified age as a significant predictor of PMV among postoperative intracerebral hemorrhage patients who survived beyond 14 days.

Comorbid chronic kidney disease was associated with a markedly increased risk of PMV (OR = 5.46). This likely reflects multiple physiologic barriers to weaning rather than a single complication. Patients with chronic kidney disease are prone to perioperative fluid retention and volume fluctuations, which can contribute to pulmonary interstitial edema, reduced lung compliance, and impaired oxygenation, making weaning more difficult (6). In addition, acid–base disturbances and anemia increase respiratory workload and reduce exercise tolerance, so even modest increases in respiratory demand may precipitate SBT failure or reintubation (7). Immune dysregulation and chronic inflammation may further increase the risk of pulmonary infection and sepsis. Once ventilator-associated infection develops, a cycle of infection, deeper sedation, limited mobility, respiratory muscle weakness, and weaning failure may prolong ventilator dependence. Evidence from long-term weaning centers also suggests lower weaning success and higher mortality among patients with renal dysfunction, consistent with our findings (8).

Lower admission GCS score was independently associated with PMV, suggesting that PMV in this postoperative population is largely driven by neurologic status. Reduced consciousness implies weakened airway-protective reflexes and ineffective cough, increasing the risk of aspiration, secretion retention, infection, and recurrent respiratory failure (9). Moreover, unstable intracranial conditions often necessitate deeper or prolonged sedation and analgesia, which can delay awakening, mobilization, and spontaneous breathing training, thereby postponing the optimal weaning window. Xiao et al. (10) similarly reported that initial GCS score is closely associated with PMV. Other studies have also shown that hematoma burden and systemic organ dysfunction at ICU admission can predict PMV, supporting a framework in which neurologic injury severity and systemic involvement jointly shape the weaning course (11).

Intraoperative drainage catheter placement was independently associated with PMV in the primary model (OR = 3.51) and remained significant in a sensitivity analysis that excluded patients who underwent cranial puncture drainage and additionally adjusted for the remaining surgical classifications (OR = 2.92, 95% CI 1.37–6.24; *p* = 0.006). This finding suggests that the observed association was not solely driven by procedure-specific catheter use in the cranial puncture drainage subgroup. Clinically, catheter placement is procedure-dependent and may also reflect greater hematoma burden, residual clot, intracranial pressure concerns, cerebral edema, or intraoperative judgment. Therefore, this variable should be interpreted as a surgery-related marker of case complexity rather than as an isolated technical exposure. In addition, intracranial catheters may increase the risk of catheter-related infection and inflammatory burden. Once intracranial or systemic infection occurs, it may lead to deeper sedation, reduced activity, and escalation of respiratory support, thereby further prolonging the ventilation course (12, 13).

Notably, tracheotomy was not an independent predictor of PMV in our model (*p* = 0.091). In practice, tracheotomy is often performed after weaning difficulty has already emerged, when prolonged airway protection is anticipated, or after repeated extubation failure, which may explain its apparent association with PMV (14). After adjustment for baseline severity (including age, admission GCS score, and intraoperative drainage catheter placement), the association was no longer significant, suggesting that tracheotomy is more likely a marker of weaning difficulty rather than an independent driver of PMV. The timing of tracheotomy may further modify its relationship with outcomes; our study did not stratify tracheotomy as “early” versus “late.” Trouillet et al. suggested that early tracheotomy may shorten ventilation duration by reducing sedation needs, improving airway care, and facilitating rehabilitation (15). Conversely, late or complication-driven tracheotomy often occurs in patients with a more complex course and may be associated with longer ventilation duration and worse outcomes (16). Future studies should evaluate timing using time-dependent approaches rather than only comparing whether tracheotomy was performed.

A prediction model based on four readily available variables—age, chronic kidney disease, admission GCS score, and intraoperative drainage catheter placement—showed moderate-to-good discrimination (AUC = 0.779) and acceptable calibration. Because these predictors are available at admission or immediately after surgery, the model may support early risk stratification in the ICU and inform resource planning and management intensity. Nevertheless, the model’s performance suggests that additional predictors remain unmeasured, particularly time-varying factors during ICU care (e.g., ventilator-associated infection, cumulative sedative exposure, changes in respiratory mechanics, trajectories of inflammatory markers, delirium, and early rehabilitation). Incorporating such dynamic variables may improve future model updating and external validation. Clinically, patients with high-risk features may benefit from proactive strategies, including structured sedation interruption and awakening protocols, enhanced airway care and aspiration prevention, early respiratory rehabilitation, and closer management of volume status, electrolytes, and infection in those with chronic kidney disease, thereby reducing the cumulative burden of complications and improving weaning outcomes.

This study identified independent predictors of prolonged mechanical ventilation (PMV) and established a 4-variable predictive model for PMV in postoperative patients with hypertensive basal ganglia spontaneous intracerebral hemorrhage (SICH). Two core lines of future research on this topic warrant further exploration: first, large-scale multi-center external validation of the predictive model to confirm its stability and generalizability in wider clinical settings; second, prospective clinical trials to evaluate whether early targeted intervention based on this risk stratification model can reduce the incidence of PMV and improve long-term clinical outcomes in this high-risk patient population.

Several limitations of this study need to be noted. First, this was a single-center retrospective cohort study, with inherent selection and information bias, which may limit the generalizability of the results. Second, we only included postoperative patients with hypertensive basal ganglia hemorrhage, so the findings cannot be directly extrapolated to conservatively treated patients or those with other types of intracerebral hemorrhage. Third, we were unable to fully adjust for all potential confounding factors affecting PMV risk due to the retrospective study design. Although we performed supplementary procedure-stratified and sensitivity analyses, residual confounding related to surgical indication, hematoma complexity, and intraoperative decision-making cannot be fully excluded. In addition, cause-specific mortality (neurological vs. non-neurological) was not consistently adjudicated in the medical records and therefore could not be analyzed reliably in the present study.

## 5 Conclusion

Among postoperative patients with spontaneous basal ganglia intracerebral hemorrhage who required mechanical ventilation and were admitted to the ICU, the incidence of PMV (≥14 days) was high. Older age, comorbid chronic kidney disease, lower admission GCS score, and intraoperative drainage catheter placement were independent predictors of PMV. A prediction model based on these four readily available preoperative and intraoperative variables showed moderate-to-good discrimination (AUC = 0.779) and acceptable calibration, and may facilitate early postoperative risk stratification, resource planning, and optimization of respiratory management.

## Data Availability

The raw data supporting the conclusions of this article will be made available by the authors, without undue reservation.
