# Supplementary material for: Predictors of prolonged mechanical ventilation after surgery for hypertensive basal ganglia intracerebral hemorrhage: a retrospective cohort study
Source: Front Med (Lausanne). 2026 Apr 9;13:1797872. doi: 10.3389/fmed.2026.1797872 (PMC13102754; doi:10.3389/fmed.2026.1797872)
Supplement: Supplementary file 1 [file Data_Sheet_1.docx]

| **Supplementary Table S1. Proportions of intraoperative drainage catheter placement according to surgical classification** | | |
| --- | --- | --- |
| Variable | Total number of cases n | Catheter placement n (%) |
| Surgical Classification[n (%)] |  |  |
| Cranial puncture drainage | 32 | 32（100.0） |
| Endoscopic hematoma evacuation | 56 | 33（62.3） |
| Craniotomy without decompressive craniectomy | 45 | 13（28.9） |
| Craniotomy with decompressive craniectomy | 40 | 22（55.0） |

Note: Data are presented as n or n (%). This table summarizes the proportion of intraoperative drainage catheter placement across surgical classifications. Because catheter placement is inherent to cranial puncture drainage and varies across other operative techniques, the observed association between catheter placement and prolonged mechanical ventilation should be interpreted in the context of surgical approach.

| **Supplementary Table S2. Comparison of surgical classification between the non-prolonged mechanical ventilation and prolonged mechanical ventilation groups after exclusion of cranial puncture drainage cases** | | | | |
| --- | --- | --- | --- | --- |
| Variable | Non-prolonged mechanical ventilation group | Prolonged mechanical ventilation group | Test statistic (χ²) | *P*-value |
| Surgical Classification (n, %) |  |  | 4.564 | 0.102 |
| Endoscopic hematoma evacuation | 41（41.4） | 15（35.7） |  |  |
| Craniotomy without decompressive craniectomy | 35（35.4） | 10（23.8） |  |  |
| Craniotomy with decompressive craniectomy | 23（23.2） | 17（40.5） |  |  |

Note: Patients who underwent cranial puncture drainage were excluded from this analysis because catheter placement is inherent to this procedure. The remaining cohort included patients undergoing endoscopic hematoma evacuation, craniotomy without decompressive craniectomy, and craniotomy with decompressive craniectomy. Data are presented as n (%). Group differences were compared using the chi-square test.

| **Supplementary Table S3. Sensitivity analysis: multivariable logistic regression of factors associated with prolonged mechanical ventilation after exclusion of patients who underwent cranial puncture drainage** | | | | | |
| --- | --- | --- | --- | --- | --- |
| Variables | B | SE | Z | P | OR (95% CI) |
| Age(years) | 0.039 | 0.015 | 2.60 | 0.009 | 1.04(1.01-1.07) |
| Chronic kidney disease | 1.44 | 0.64 | 2.25 | 0.025 | 4.22(1.20-14.80) |
| GCS score | -0.191 | 0.061 | -3.13 | 0.002 | 0.83(0.73-0.93) |
| Intraoperative drainage catheter  placement | 1.07 | 0.39 | 2.74 | 0.006 | 2.92(1.37-6.24) |
| Surgical classification (overall) |  |  |  | 0.148 |  |
| Constant | -1.31 | 1.06 | -1.24 | 0.216 |  |

Note: Patients who underwent cranial puncture drainage were excluded from this sensitivity analysis to reduce procedure-related confounding, because catheter placement is inherent to this operative technique. A multivariable logistic regression model was fitted in the restricted cohort, including age, chronic kidney disease, GCS score, intraoperative drainage catheter placement, and surgical classification. Data are presented as regression coefficients (B), standard errors (S.E.), z values, P values, and odds ratios (ORs) with 95% confidence intervals (CIs). The P value for surgical classification represents the overall effect of this variable in the model.
